# Breastfeeding and the major fermentation metabolite lactate determine occurrence of *Peptostreptococcaceae* in infant feces

**DOI:** 10.1080/19490976.2023.2241209

**Published:** 2023-08-18

**Authors:** Lucía Huertas-Díaz, Rikke Kyhnau, Eugenio Ingribelli, Vera Neuzil-Bunesova, Qing Li, Mari Sasaki, Roger P. Lauener, Caroline Roduit, Remo Frei, CK-CARE Study Group, Ulrik Sundekilde, Clarissa Schwab

**Affiliations:** a Department of Biological and Chemical Engineering, Aarhus University, Aarhus, Denmark; b Department of Food Science, Aarhus University, Aarhus, Denmark; c Department of Microbiology, Nutrition and Dietetics, Czech University of Life Sciences Prague, Prague, Czech Republic; d University Children’s Hospital Zürich, Zürich, Switzerland; e Christine Kühne-Center for Allergy Research and Education (CK-CARE), Davos, Switzerland; f Children’s Hospital St. Gallen, St. Gallen, Switzerland; g Department of Paediatrics, Inselspital, University of Bern, Bern, Switzerland

**Keywords:** Exclusively breastfeeding, gut microbiota, lactate, *Peptostreptococcaceae*, HMO fermentation metabolites

## Abstract

Previous studies indicated an intrinsic relationship between infant diet, intestinal microbiota composition and fermentation activity with a strong focus on the role of breastfeeding on microbiota composition. Yet, microbially formed short-chain fatty acids acetate, propionate and butyrate and other fermentation metabolites such as lactate not only act as substrate for bacterial cross-feeding and as mediators in microbe–host interactions but also confer antimicrobial activity, which has received considerably less attention in the past research. It was the aim of this study to investigate the nutritional–microbial interactions that contribute to the development of infant gut microbiota with a focus on human milk oligosaccharide (HMO) fermentation. Infant fecal microbiota composition, fermentation metabolites and milk composition were analyzed from 69 mother-infant pairs of the Swiss birth cohort Childhood AlleRgy nutrition and Environment (CARE) at three time points depending on breastfeeding status defined at the age of 4 months, using quantitative microbiota profiling, HPLC-RI and ^1^H-NMR. We conducted *in vitro* fermentations in the presence of HMO fermentation metabolites and determined the antimicrobial activity of lactate and acetate against major *Clostridiaceae* and *Peptostreptococcaceae* representatives. Our data show that fucosyllactose represented 90% of the HMOs present in breast milk at 1- and 3-months post-partum with fecal accumulation of fucose, 1,2-propanediol and lactate indicating fermentation of HMOs that is likely driven by *Bifidobacterium*. Concurrently, there was a significantly lower absolute abundance of *Peptostreptococcaceae* in feces of exclusively breastfed infants at 3 months. *In vitro*, lactate inhibited strains of *Peptostreptococcaceae*. Taken together, this study not only identified breastfeeding dependent fecal microbiota and metabolite profiles but suggests that HMO-derived fermentation metabolites might exert an inhibitory effect against selected gut microbes.

## GRAPHICAL ABSTRACT

**Figure:**
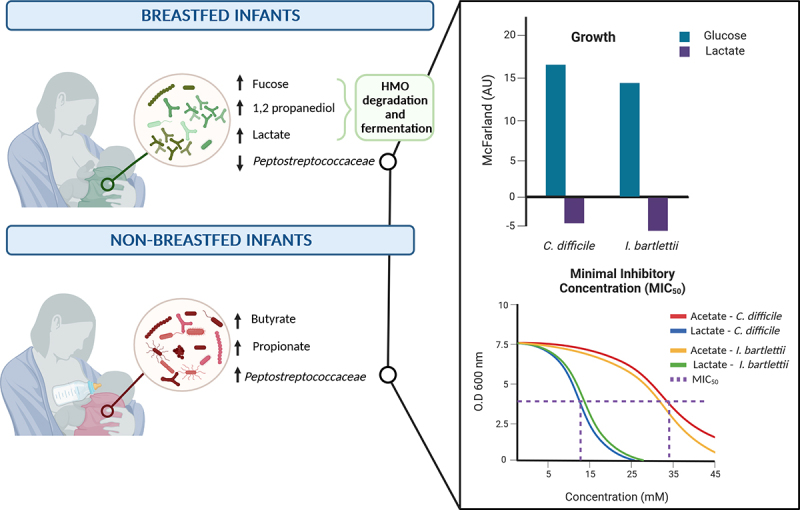

## Introduction

The infant gut starts to be colonized from birth through vertical transmission, and microbes are received from the environment. The human gut microbial ecosystem is highly dynamic with temporarily changing nutrient supplies.^1^ Such nutrient alterations are especially distinctive during the first year of life, when a physiologically developing gut system hosting a community of intestinal microbes is exposed to two major dietary transition stages. The first transition takes place soon after birth with the start of the lactation period and the feeding of breast milk. Breast milk mainly contains lipids (3.2 to 3.6 g dL^−1^), proteins (0.9 to 1.2 g dL^−1^), lactose (6.7 to 7.8 g dL^−1^), human milk oligosaccharides (HMOs) (0.5 to 2 g dL^−1^) and lower amounts of metabolites such as butyrate.^2–5^ There are up to 200 structurally and compositionally different HMOs, which are not digested by the host and consist of five monosaccharides: D-glucose, D-galactose, L-fucose, N-acetyl-D-glucosamine and sialic acid.^3^ The core structure is lactose that can be elongated with lacto-N-biose or N-acetyllactosamine and fucosylated and/or sialylated at terminal positions.^6^ Milk of mothers that lack the fucosyltransferase 2 encoding gene (*fut2*) (FUT2 non-secretor status) do not secrete α1–2 fucosylated HMO. Non-secretor status has been linked to an overall lower HMO content.^7^ During the breastfeeding period, *Bifidobacterium* spp. of the phylum *Actinomycetota* (formerly known as *Actinobacteria)* are generally the major bacterial taxa as some species are adapted to degrade and cross-feed on HMO and HMO degradation products. *Bifidobacterium* spp. including *Bifidobacterium longum* subsp. *infantis* digest HMOs intracellularly, while *Bifidobacterium bifidum* hydrolyses HMOs extracellularly enabling microbial cross-feeding. Major fermentation metabolites of *Bifidobacterium* HMO metabolism are acetate, lactate, formate and the fucose degradation product 1,2-propanediol (1,2-PD).^8–10^ Colonization by a low number of butyrate and propionate producers during the first months of life relates to low fecal levels of propionate and butyrate that have been observed in several studies.^10–12^ The earliest butyrate producers were suggested to be members of the endospore forming *Clostridiaceae* and *Peptostreptococcaceae*.^12^

In non-breastfed infants, *Bifidobacterium* spp. often represent a major proportion of the gut microbiota, α-diversity of fecal microbiota of non-breastfed infants is often higher than that of breastfed infants.^13–15^ Formula feeding has been associated with higher abundance of *Clostridium* spp. and of *Clostridioides difficile* compared to breastfed infants.^16,17^

The second nutrient transition occurs during the weaning period with the introduction of solid foods containing structurally and compositionally more complex carbohydrates.^18^ During weaning, the gut microbiota diversifies taxonomically and functionally^19^ leading to a microbiota dominated by the phyla *Bacteroidota* and *Bacillota*.^20^ The increase in abundance of butyrate producing taxa including *Faecalibacterium prausnitzii*, the *Roseburia* spp,*/Eubacterium rectale* group and *Anaerobutyricum hallii* during weaning has been related to higher fecal butyrate levels.^11,21^ Fecal fermentation profiles after weaning are concurrently characterized by higher propionate levels compared to pre-weaning.^11,12,21^

Previous studies indicated a strong relationship between infant diet, fecal microbiota composition and fermentation activity represented by fecal short chain fatty acids (SCFA) and lactate.^20,22^ Acetate, propionate and butyrate and other major fermentation metabolites including lactate not only act as substrate for bacterial cross-feeding and as mediators in microbe–host interactions but also have antimicrobial activity.^23^ In food fermentation systems, the antimicrobial activity of lactate and other SCFA contribute to biopreservation effects through the inhibition of sensitive microbes, such a bioactivity has been much less investigated in the gut microbial ecosystem^24^.

The aim of this study was to elucidate the impact of breastfeeding status on fecal fermentation metabolites and their effect on specific taxa during the first year of life.

## Results

### Baseline breastfeeding and delivery characteristics of infants

**Figure 1.**
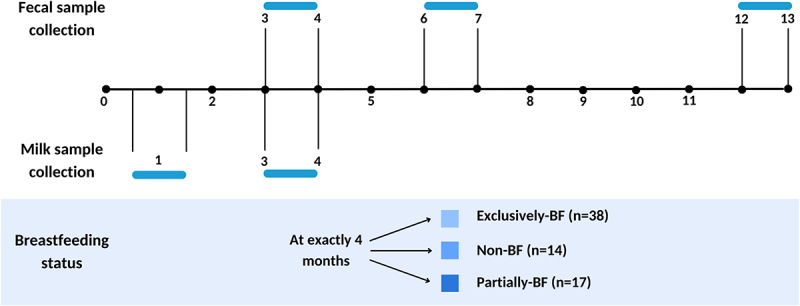
Timeline of sample collection and division by breastfeeding status.

Notes: Children were divided by their breastfeeding status at exactly 4 months into Exclusively-breastfed (BF), Non-BF and Partially-BF. Fecal samples were collected between 3–4 months (termed as 3 months throughout the manuscript), 6–7 months (termed as 6 months), and 12–13 months (termed as 12 months) (blue intervals). Breastmilk samples were collected at 1 month (±1 week) and at 3–4 months (termed as 3 months). n: denotes the number of participants in each group.

Milk and fecal samples of 69 mothers and their infants, respectively, were collected in the context of the ongoing Swiss birth cohort ‘Childhood AlleRgy nutrition and Environment’ (CARE) between 2016 and 2019. The major aim of the CARE study has been to determine the relationship between nutrition, the gut microbiota, and the development of allergic diseases such as atopic dermatitis in early life. The 69 mother-infant pairs were grouped depending on their breastfeeding status reported at 4 months of age, which is the recommended length of breastfeeding only in Switzerland (www.blv.admin.ch). Infants were grouped as exclusively-breastfed (BF) (*n = 38*) if they only received breast milk at 4 months, while partially-BF (*n = 17*) infants obtained breastmilk and complementary formula (mixed breastfeeding). Non-BF infants (*n = 14*) only received formula at 4 months (Figure 1).

Based on dietary diaries collected until 1 year of age, the infants in this cohort were mixed breastfed for a median of 25 weeks (interquartile range (IQR) 11–32 weeks). *N* = 25 mothers (36%) mixed breastfed at 52 weeks. The median duration of only breastfeeding was 20 (IQR 17–21) weeks for infants classified as exclusively-BF, 0 (IQR 0–1.75) weeks for non-BF, and 10 (IQR 3–15) weeks for partially-BF groups (Table 1). Exclusively BF infants (defined at 4 months) were mixed breastfed for a length of 29 (IQR 17–32) weeks, while non-BF and partially-BF infants, defined at the age of 4 months, were mixed breastfed for 7.5 (IQR 2–9.8) and 34 (IQR 21–37) weeks, respectively (Table 1).

**Table 1.** Baseline characteristics of the infants based on breastfeeding status.

		Breastfed (BF) status (weeks; Median and IQR)^a^			
		Exclusively-BF (*n = 38*)	Non-BF^b^ *(n = 14*)	Partially-BF (*n = 17*)	p-value
Length of breast feeding^c^	Only breastfeeding	20 (17–21)	0 (0–1.75)	10 (3–15)	***
	Mixed breastfeeding	29 (17–32)	7.5 (2–9.75)	34 (20.5–37)	***
Delivery mode	Vaginally (*n = 54*)	28 (74%)	12 (86%)	14 (82%)	n.s
	C-section (*n = 15*)	10 (26%)	2 (14%)	3 (18%)	n.s

Infants were divided in exclusively-, partially- or non-breastfed (BF) infants depending on the breastfeeding status at 4 months, which is the minimum length of only breastfeeding recommended in Switzerland. Shown are the total length of only and mixed breastfeeding for exclusively-BF, Non-BF and partially-BF based on diaries collecting data during the first year of life. *n* = number of infants. Significant differences for length of breastfeeding and for delivery mode distribution are presented as: ‘***’ *p* < 0.001 ‘**’ 0.01 ‘*’ 0.05 ‘n.s’ no significance.

^a^Non-BF infants account for infants that were not breastfed at the cutoff point of 4 months. Infants from this group could have been breastfed before this point.

^b^Only considered the information from the diaries that were collected during the first year.

^c^Presented in absolute numbers and percentage for delivery mode and in weeks average, median and interquartile range (IQR) for the duration of breastfeeding.

There was no significant difference in delivery mode for exclusively-BF infants (74% of vaginal and 26% C-section delivery), partially-BF (86% and 14%) and non-BF (82% and 18%) infants (Table 1). The median of mixed breastfeeding was 25 and 27 weeks for vaginal and C-section delivered infants, respectively.

### Carbohydrates and metabolites present in breast milk

To determine the composition and amount of HMOs, lactose, SCFA and lactate that were present in breast milk, we used proton nuclear magnetic resonance, ^1^H-NMR. To cover two stages during lactation, samples collected at 1 month (±1 week) and 3–4 months (termed as 3 months) were analyzed. At 1 month, 96.5% of the infants were either only or mixed breastfed, while at 3 months, 79.7% of the infants received breastmilk.

The most abundant HMOs were 2-fucosyllactose (2-FL) and 3-fucosyllactose (3-FL) presenting together 90% of the HMOs (Suppl. Table S1). 2-FL was present in more than 76% of the samples at 1 month and over 83% at 3 months. Based on the presence of 2-FL, 80% of the mothers were identified as FUT2-secretors (Suppl. Table S1). 3-FL was significantly higher (*p < 0.05*) in non-secretor compared to FUT2-secretor (Suppl. Figure S1). Concentrations ranged between 1148.4–5255.9 μM for 2-FL and 148.5–5552.9 μM for 3-FL at 1 month and between 1022.3–4577.1 μM (2-FL) and 387.1–7493.6 μM (3-FL) at 3 months (Suppl. Figure S1). At 3 months, the median of 2-FL (2069.3 μM, IQR 1628.6–2483.3 μM) was significantly lower compared to 1 month (2781.2 μM, IQR 2121.1–3453.8 μM, *p < 0.05*) (Suppl. Figure S1).

Lacto-N-fucopentaose I (LNFP I) was detected in all samples, while Lacto-N-difucohexaose (LDFT), Lacto-N-difucohexaose (LNDFH I and II) and lacto-N-fucopentaose V (LNFP V) were present in 57–100% of the samples (Suppl. Table S1). With the exception of 3-FL, LDFT and LNDFH II, all HMOs differed (*p < 0.05*) in concentration between time points (Suppl. Figure S1). From 1 to 3 months, median levels of Lacto-N-difucohexaose I (LNDFH I) (1194.9; 598 μM) and LNFP I (511.2; 202.8 μM) significantly (*p < 0.05*) decreased (Suppl. Figure S1). The only HMO that increased its concentration significantly (*p < 0.05*) from 1 to 3 months was LNFP V (300.5; 619.2 μM).

The sialic HMO 3-sialyllactose (3-SL) (187.11; 133.7 μM), 6-sialyllactose (6-SL) (80; 25.7 μM), and disialyllacto-N-tetraose (DSLNT) (206.6; 81.5 μM) were present in all samples at significantly higher levels (*p < 0.05*) at 1 compared to 3 months (Suppl. Figure S1). At 1 month, 3-SL was significantly higher (*p < 0.05*) in non-BF and exclusively-BF compared to partially-BF infants (Suppl. Table S1).

Lactose was recovered from all samples (125.2; 135.6 mM) with a significant increase in levels (*p < 0.05*) between 1 and 3 months (Suppl. Figure S1 and Table S1). The SCFA acetate, butyrate, and lactate, were present in all breast milk samples (Suppl. Table S2). Formate was detected in 77% of the samples at 1 month and in 74% at 3 months. Acetate levels ranged from 25.6 to 42.9 μM with no difference between 1 and 3 months (Suppl. Figure S2). The median level of butyrate at 1 month was 60.6 μM, which significantly increased (*p < 0.05*) to 228.4 μM at 3 months (Suppl. Figure S2). At 1 and 3 months, formate concentration from 15.5 to 14.5 μM, while lactate concentrations ranged from 137.4 to 154.8 μM (Suppl. Figure S2 and Table S2).

These data show that breast milk is not only a source of HMO but also a vehicle for SCFA and lactate.

### Infant fecal microbiota composition

Next, we next re-analyzed the data generated by Appert *et al*. ^12^ for the same cohort of samples collected within the months that are recommended for exclusive breastfeeding (3–4 months, termed 3 months throughout the manuscript), after the introduction of solid foods at 6–7 months (termed 6 months) and 12–13 months (termed 12 months). Fecal microbiota composition was determined based on breastfeeding status at 4 months using quantitative microbiota profiling based on combined 16S rRNA gene sequencing data (accessible at PRJNA616703) and quantitative PCR as provided by Appert *et al*. ^12^

**Figure 2.**
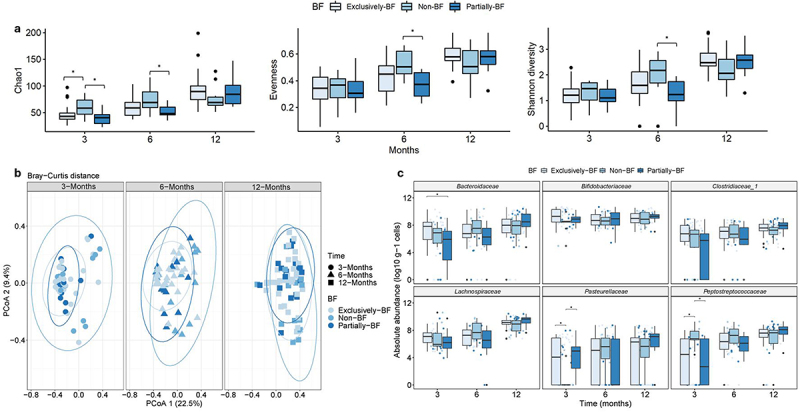
Fecal concentrations of fucose and fermentation metabolites.

Notes: Analysis of microbial composition was based on 16S RNA gene data.^12^ Samples were collected between 3–4 months (termed as 3 months), 6–7 months (termed as 6 months), and 12–13 months (termed as 12 months). a) Alpha-diversity at different time points depending on breastfeeding status of the infants (Chao1 index, evenness, and Shannon diversity). b) Beta-diversity based Principal Coordinate Analysis (PCoA) plots calculated from the Bray-Curtis distance based on time point and breastfeeding status. c) Absolute abundance based on quantitative microbiota profiling of the most relevant families according to breastfeeding status and time point. Significant differences are indicated as: ‘***’ 0.001 ‘**’ 0.01 ‘*’ 0.05. Non-BF: non-breastfed; Partially-BF: partially breastfed; Exclusively-BF: exclusively breastfed.

Alpha-diversity indices increased from 3 to 12 months in all three breastfeeding groups (Figure 2a). At 3 months, richness based on Chao1 was 1.4 times higher for the non-BF group compared to the exclusively-BF (*p < 0.05*) and partially-BF (*p < 0.05*) groups (Figure 2a). At 6 months, Chao1, evenness and Shannon diversity of partially-BF were significantly lower (*p < 0.05*) than of non-BF, while at 12 months there was no difference in α-diversity indices and breastfeeding status (Figure 2a).

Beta-diversity was evaluated using the Bray-Curtis distance. In principal coordinates analysis (PCoA), PcoA 1 captured 22.5% of the data while PcoA 2 explained 9.4% (Figure 2b). At 3 and 6 months, the majority of samples of exclusively-BF and partially-BF clustered together, while the samples collected at 12 months were more dispersed similar to the samples collected from non-BF infants (Figure 2b). There was a significant difference (*p < 0.05*) between the β-diversity distances regarding time (at 3, 6 and 12 months) or breastfeeding status (between exclusively, partially and non-BF), but there were no differences if both variables were combined (Figure 2b).

Based on 16S rRNA gene sequencing, most reads (>55%) were assigned to *Bifidobacteriaceae* at 3 months (Suppl. Figure S3). *Enterobacteriaceae* and *Bacteroidaceae* contributed 13.6% and 7.8%, respectively, in exclusively-BF. Major families in feces of non-BF were *Lachnospiraceae* (12.9%), *Peptostreptococcaceae* (9%) and *Enterococcaceae* (4.6%), whose abundance was <2% in exclusively or partially-BF infants. At 1 year of age, the three major bacterial families were *Bifidobacteriaceae* (22–36%), *Lachnospiraceae* (23–27%) and *Bacteroidaceae* (1–6%) in all three breastfeeding groups (Suppl. Figure S3).

Next, we used quantitative microbiota profiling to determine the absolute abundance of major bacterial families in the different breastfeeding groups (Figure 2c). The absolute abundance of *Bifidobacteriaceae* remained constant during the first year for all the breastfeeding groups with a median of 9.0 (IQR 8.2–9.7) log_10_ cells g^−1^ for exclusively-BF, 9.1 (IQR 8.6–9.5) log_10_ cells g^−1^ for partially-BF and 8.5 (IQR 8.1–9.4) log_10_ cells g^−1^ for non-BF.

**Figure 3.**
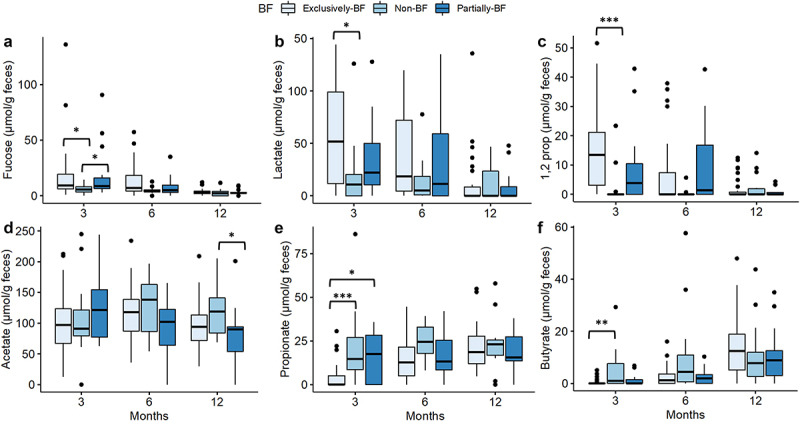
Fecal concentrations of fucose and fermentation metabolites.

Notes: Concentrations (μmol g^−1^ feces) were determined at three time points 3–4 months (termed as 3 months), 6–7 months (termed as 6 months), and 12–13 months (termed as 12 months) and were separated according to the different breastfeeding conditions at 4 months of a) fucose, b) lactate, c) 1,2 propanediol (1,2-prop), d) acetate, e) propionate and f) butyrate. Significant differences shown: ‘***’ 0.001 ‘**’ 0.01 ‘*’ 0.05. Non-BF: non-breastfed; partially-BF: partially breastfed; exclusively-BF: exclusively breastfed.

Cell counts of *Bacteroidaceae* were significantly higher for exclusively-BF (7.8 log_10_ cells g^−1^) compared to partially-BF (6.0 log_10_ cells g^−1^) (*p < 0.05*) at 3 months (Figure 2c). At 3 months, there was a significantly higher abundance of *Pasteurellaceae* in exclusively-BF (4.1 log_10_ cells g^−1^ (*p < 0.05*)) and partially-BF (5.0 log_10_ g^−1^ (*p < 0.05*) compared to non-BF (0 log_10_ cells g^−1^). Abundance of *Peptostreptococcaceae* was significantly (*p < 0.05*) lower for exclusively-BF (4.5 log_10_ cells g^−1^) and partially-BF (2.7 log_10_ cells g^−1^) compared to non-BF (6.8 log_10_ cells g^−1^) infants (Figure 2c). At 12 months, no significant difference was observed for abundance of *Peptostreptococcaceae* between groups (7.7 log_10_ cells g^−1^ median). The absolute abundance of *Clostridiaceae* increased over time from 6.8 to 7.6 log_10_ cells g^−1^ in exclusively-BF, 5.8 to 7.9 log_10_ cells g^−1^ in partially-BF and from 6.7 to 7.3 log_10_ cells g^−1^ in non-BF. The absolute abundance of *Lachnospiraceae* was around 5.9–7.2 log_10_ cells g^−1^ at 3 months and increased to 7.9–9.6 log_10_ cells g^−1^ at 12 months (Figure 2c).

Together, these data show that breastfeeding status was associated with higher abundance of bacterial families *Bacteroidaceae*, *Pasteurellaceae* and lower counts of *Peptostreptococcaceae* especially when samples from 3 months old infants were considered.

### Fecal fermentation metabolite and fucose profiles

Fecal levels of fucose and of fermentation metabolites that were generated and presented by Appert *et al*. ^12^ and Sasaki *et al*.^25^ for the same cohort were re-analyzed to establish whether there was an association between the breastfeeding status and intestinal microbial fermentation activity.

Fucose was present in the majority of fecal samples (>92%) at 3 months (Suppl. Table S3); fecal levels were higher for exclusively-BF (*p < 0.05*) or partially-BF (*p < 0.05*) compared to non-BF (Figure 2a). At 3 months, lactate was detected in 80% and 62% of the feces of the BF- and non-BF infants, respectively (Suppl. Table S3). Fecal lactate levels of exclusively-BF (median 52 μmol g^−1^ feces) were significantly higher than of the non-BF (11 μmol g^−1^, *p < 0.05*) group (Figure 2b). At 3 months, there was a significant difference in the occurrence of 1,2-PD in feces of exclusively-BF (83%), partially-BF (63%) and non-BF (23%) infants (*p < 0.05*) (Suppl. Table S3). Median levels of 1,2-PD were higher (14 μmol g^−1^) for the exclusively-BF compared to the non-BF infants (0 μmol g^−1^ (*p < 0.05*)) (Figure 2c).

Acetate was the major SCFA present in the feces with a significant difference (*p < 0.05*) between non-BF and partially-BF infants at 12 months (Figure 2d). At 3 months, the occurrence of propionate and butyrate in exclusively-BF infants (41% and 21%) was significantly lower (*p < 0.05*) compared to non-BF (92% and 62%, Suppl. Table S3). Median levels of fecal propionate were significantly higher for both partially (*p < 0.05*) and non-BF (*p < 0.001*) compared to exclusively-BF at 3 months (Figure 2e), while butyrate levels were significantly higher in non-BF compared to exclusively-BF (*p < 0.01*).

Taken together, these data show that breastfeeding status had a strong impact on fecal fermentation profiles especially at 3 months. There was an inverse relationships of lactate, and butyrate and propionate, while fucose and its major metabolite 1,2-PD accumulated together with lactate in feces of breastfed infants.

### Relationship of breast milk composition, fecal microbiota abundance and fermentation metabolites

To identify relationships between quantitative breastfeeding data and microbiota abundance fecal metabolites levels, we performed a factor analysis for mixed data (FAMD) of fecal and milk samples collected at 3 months.

**Figure 4.**
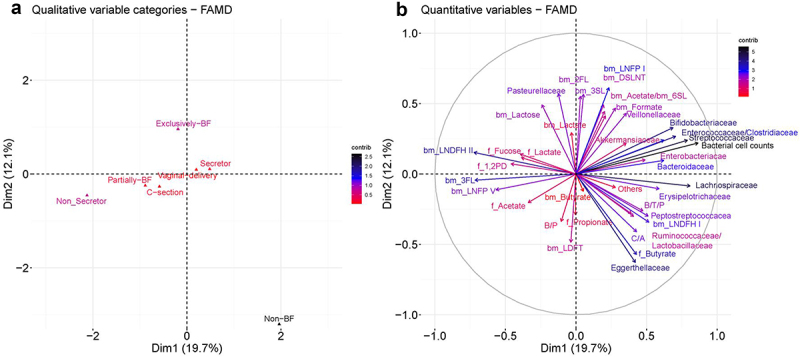
Relationship of fecal microbiota composition, fermentation metabolites and milk composition with delivery mode, secretor phenotype and breastfeeding status.

Notes: Factor analysis for mixed data (FAMD) including categorical variables such as delivery mode, breastfeeding status, secretor phenotype and quantitative data such as absolute abundance of the families derived from quantitative microbiota profiling, fecal fermented metabolites (f_) and breastmilk metabolites (bm_) at 3 months.

The first dimension explained 19.7% of the data, while the second dimension explained 12.1% (Figure 4). The variable ‘exclusively-BF’ was related to ‘*Pasteurellaceae’*, and fecal concentrations of ‘lactate’, ‘1,2-PD’ and ‘fucose’ and levels of ‘lactose’, ‘3SL’, ‘DSLNT’ and ‘2FL’ in milk; while variable ‘Non-BF’ samples was linked to fecal ‘butyrate’ and ‘propionate’, as well as abundance of ‘*Peptostreptococcaceae*’, ‘*Erysipelotrichaceae*’, ‘*Eggerthellaceae*’, ‘*Lactobacill-aceae’* and ‘*Ruminococcaceae*’ family (Figure 4). ‘Delivery mode’, ‘Partially-BF’ or ‘secretor phenotype’ did not have any impact in the quantitative parameters in these two dimensions (Figure 4).

### Impact of fucose, 1,2-PD and lactate on growth and metabolic activity of selected *Peptostreptococcaceae* and *Clostridiaceae*

Since the abundance of the *Peptostreptococcaceae* was higher for the non-BF group compared to exclusively and partially-BF at 3 months, and there was an inverse relationship with BF status and fecal lactate, fucose and 1,2-PD levels (Figures 2c, 4), we investigated the response of selected *Peptostreptococcaceae* to the presence of compounds that were accumulated in feces of BF infants, i.e., the HMO component fucose, and the fermentation intermediates lactate and 1,2-PD and compared the response to members of the *Clostridiaceae*. *Clostridiaceae* was selected as a phylogenetically and functionally closely related taxon that potentially also contributes to early butyrate production in the infant gut.^12,26^

Based on the data generated by Appert *et al*.^12^ and additional blast analysis, the majority of Amplicon Sequence Variants (ASVs) assigned to the family *Peptostreptococcaceae* were *C. difficile* (81.7%) and *Intestinibacter bartlettii* (13.2%) (Suppl. Table S4). The majority of *Clostridiaceae* ASVs were assigned to *Clostridium sensu stricto*, i.e. *Clostridium perfringens* (61.5%), *Clostridium butyricum* (16.3%) and *Clostridium paraputrificum* (10.5%) with lower proportions of *Clostridium baratii* and *Clostridium neonatale* (0.5–1.3%) (Suppl. Table S4). We obtained representative strains from commercial and laboratory culture collections and conducted growth assays in Hungate tubes using anaerobically prepared modified yeast-extract casitone medium without fatty acids (YC, Duncan *et al*.)^27^. Measurements of turbidity were performed after 0 and 24 h of incubation at 37°C, substrate utilization and metabolite formation were determined using HPLC-RI. The increase in turbidity in YC medium without carbon source (0.2–3.8 McFarland units, AU) and corresponding metabolites were subtracted.

**Figure 5.**
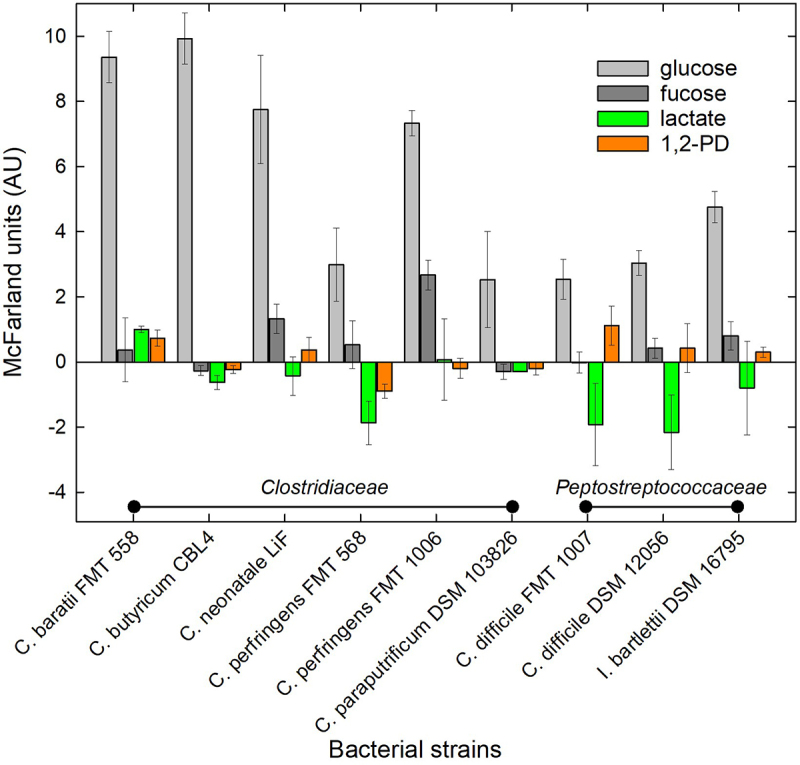
Turbidity of selected strains of *Clostridiaceae* and *Peptostreptococcaceae* in YC medium.

Notes: Turbidity (in McFarland units (AU)) was determined after 24 h grow of different strains in presence of yeast extract casitone medium (YC) supplied with glucose (51 mM), fucose (45 mM), lactic acid (55 mM), 1,2-propanediol (1,2-PD) (42.3 mM). Shown are mean and standard deviation from at least three independent biological replicates. Background growth from yeast-extract casitone (YC) medium in the absence of added carbon source was subtracted from each measurement.

**Figure 6.**
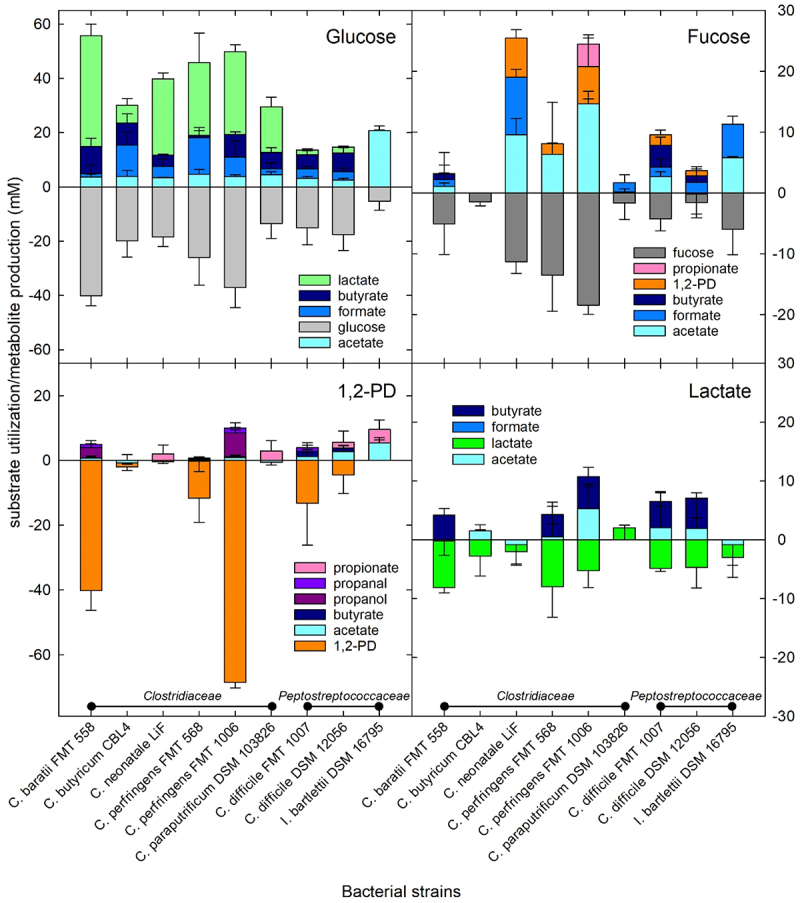
Substrate utilization and metabolite production of selected HMO components and fermentation metabolites.

Notes: Substrate utilization and metabolite production in mM from different strains in presence of YC-glucose (51 mM), fucose (45 mM), lactate (55 mM), 1,2-propanediol (1,2-PD) (42.3 mM). Shown are mean and standard deviation from three biological replicas. Background growth from yeast-extract casitone (YC) medium in the absence of added carbon source was subtracted from each measurement.

All strains grew in the presence of glucose (Figure 3) producing mostly lactate, butyrate, formate and acetate (*Clostridiaceae*) or acetate, butyrate and formate (*Peptostreptococcaceae*) (Figure 4). There was an increase in turbidity of *C. neonatale* LiF, *C. perfringens* FMT 1006, *C. difficile* DSM 12056 and *I. bartlettii* DSM 16795 when grown in the presence of fucose concurrently, fucose was used and mainly acetate, formate, and 1,2-PD were formed (Figures 5 and 6). *C. perfringens* FMT 1006 and *C. difficile* DSM 12056 and FMT 1007 produced additional propionate and butyrate (Figure 4). In the presence of lactate, turbidity only increased for *C. baratii* FMT 558, while it did not change or became lower for all other strains (Figure 3). Butyrate (3.3–1.3 mM) and/or acetate (9.5–4.5 mM) were formed by *C. baratii* FMT 558, *C. butyricum* CBL4, *C. perfringens* FMT 568 and FMT 1006, and *C. difficile* DSM 12056 and FMT 1007 (Figure 4). In the presence of 1,2-PD, there was little change in turbidity, nevertheless, *C. baratii* FMT 558 and *C. perfringens* FMT 1006 utilized about 50% and 100% of the supplied 1,2-PD (Figures 5 and 6).

These data indicate the overall capacity of infant-associated species of *Clostridiaceae* and *Peptostreptococcaceae* species to utilize fucose and 1,2-PD derived from fucosylated HMO for growth, and additionally suggest that lactate might be inhibitory toward strains of *C. perfringens*, *C. difficile* and *I. bartlettii*.

### Antimicrobial activity of acetic and lactic acid

As there was lower turbidity during growth in YC-lactic acid (55 mM) *in vitro*, we determined the antimicrobial activity of lactic acid against selected strains of *Peptostreptococcaceae* (*C. difficile* DSM 12056 and *I. bartlettii* DSM 16795) and *Clostridiaceae* (*C. perfringens* FMT 568 and FMT 1006) using broth dilution assays in 96-well microtiter plates at strict anaerobic conditions. For comparison, acetic acid, the most abundant SCFA in feces, was included in the assay. The pH of the Wilkins-Chalgren medium was adjusted to pH 5.5 and 6.2, maximal concentrations of acetic and lactic acid were 90 mM. Plates were incubated at anaerobic conditions at 37°C for 24 h until optical density was measured at 590 nm. Data were fit in a sigmoidal equation (four logistic parameters) used to calculate the minimal inhibitory concentration reducing the final density to 50% (MIC_50_).

Data could only be fit by the sigmoidal equation at pH 5.5 for *C. difficile* DSM 12056 and *I. bartlettii* DSM 16795, the MIC_50_ of lactic acid for *C. difficile* DSM 12056 and *I. bartlettii* DSM 16795 was 25 mM, while the MIC_50_ was >90 mM with acetic acid (Suppl. Figure S4). For *C. perfringens* FMT 568 and FMT 1006, the MIC_50_ toward acetic or lactic acid could not be determined, as turbidity was only reduced to 89.0 ± 3.4% and 43.1 ± 17.7%, or 51.6 ± 9.6% and 47.6 ± 13.0% in the presence of 90 mM acid, respectively, compared to controls (Suppl. Figure S4).

At pH 6.2, *C. difficile* DSM 12056 and *I. bartlettii* DSM 16795 reached 87.4 ± 19.8% and 54.1 ± 13.4% when grown with 90 mM acetic acid compared to controls, with lower maximum turbidity 34.9 ± 9.3% and 17.4 ± 8.4% when 90 mM lactic acid was present (Suppl. Figure S4). Turbidity of both *C. perfringens* with 90 mM acetic and lactic acid was 67.7 ± 12.4 and 49.6 ± 07.4% compared to positive controls. All strains reached lower optical density when grown in pH 5.5 compared to pH 6.2 (Suppl. Figure S4). There was no synergistic activity of lactic and acetic acid (data not shown).

In summary, the strains of *Peptostreptococcaceae* were more sensitive toward lactate than strains of *Clostridiaceae* at the tested conditions, and acetate had less antimicrobial activity than lactate at standardized pH.

## Discussion

During the first year of life, the infant gut microbiota changes in composition with a major gain in functional capacity with the introduction of solid food during weaning. Infant gut microbiota composition has been shown to be influenced by parameters including the mode of delivery, gestation age, the use of antibiotics, and the type of feeding.^19^ Our data showed that breastfeeding was associated with distinctive fecal fermentation metabolite profiles that related to higher abundance of *Peptostreptococcaceae* in infants that were not breastfed. *In vivo* observations together with *in vitro* experiments suggested that lactate might play an inhibitory role in the growth of members of the *Peptostreptococcaceae* family during exclusive breastfeeding.

### Fermentation of fucosylated HMO characterizes fecal fermentation profiles

2-FL and 3-FL were the major HMO found in breast milk in agreement with previous studies.^7,28–30^ 2-FL represented 30% of the HMO content of secretor mothers, which is the major phenotype in Europe accounting for almost 80% of the mothers.^29,31,32^ A similar proportion was observed for this Swiss cohort.

Infant-associated *Bifidobacterium* spp. are able to degrade and cross-feed on fucosylated HMO.^33^ Strains of *B. bifidum* liberate the fucose extracellularly, fucose that is not used in microbial cross-feeding might account for the levels of free fucose observed in feces.^34^

Strains of the species *B. longum* subsp. *infantis, B. breve*, and *B. kashiwanohense* ferment fucosyllactose and fucose producing acetate, formate, lactate and 1,2-PD.^8,10,35^ Exclusively-BF infants had higher fecal levels of both lactate and 1,2-PD compared to non-BF infants in agreement with other cohorts.^36–38^ These results indicate that the presence of fucose, 1,2-PD and lactate was directly related to breastfeeding. These compounds are products of *Bifidobacterium*-driven metabolism of the most prominent HMO 2-FL and 3-FL and might also be released by other HMO utilizers including *Bacteroidaceae*.^33,39^ It is currently under debate whether HMOs are possibly supplied in surplus as spent HMOs were recovered in feces with concentrations decreasing during the first months of breastfeeding,^40^ or whether fecal recovery of HMO depends on the abundance of certain strains of *B. longum* subsp. *infantis*.^41^

### Without nutritional restrictions during breastfeeding, microbiota profiles and fecal fermentation metabolites became more diverse

Breastfeeding has been frequently associated with a lower α-diversity compared to formula fed infants and to a dominance of *Bifidobacterium*.^13,22,42^ With the cessation of breastfeeding due to the introduction of formula or solid foods, the competitive advantage of HMO utilizers decreases, which leads to higher fecal microbial taxonomic diversity.^19,37,38^ In non-breastfed infants and after weaning, the microbial community became more diverse with a concurrent increase in fecal propionate and butyrate levels.^37,38^

During breastfeeding, fecal levels of butyrate and propionate remained low despite the potential of some functional groups to use the fermentation intermediates lactate or 1,2-PD and to produce butyrate and propionate from HMOs. As an example, the fermentation metabolite 1,2-PD can be metabolized into propionate by gut microbes such as *Anaerobutyricum hallii, Blautia obeum, Ruminococcus gnavus* and *Flavonifractor plautii* and seems to be available in excess, yet these species were low abundant at 3 months leading to the accumulation of 1,2-PD in feces.^12,43,44^ Similarly, the butyrate producing *Roseburia hominis* or *Roseburia inulivorans* can degrade and utilize HMO but were only present at low abundance (3–4 log_10_ cells g^−1^ feces) before weaning.^12,45^

Among the early detected succinate/propionate producers were members of the *Bacteroidota* with strains of the species *B. fragilis* and *B. vulgatus* being able to metabolize HMOs.^19,46^ In agreement, *Bacteroides* and *Prevotellaceae* were more abundant in exclusively-BF infants in this study, but were still present at significantly lower levels than *Bifidobacterium* possibly due to a higher competitiveness of *Bifidobacterium* spp. in utilizing HMOs with a low degree of polymerization.^47^ Early colonizing butyrate producers in the infant gut were *Clostridiaceae* and *Peptostreptococcaceae*, abundance of the last-mentioned was lower in breastfed infants.

Schwab^18^ recently suggested that the ability to use HMOs as alternative or secondary substrate might support the colonization or seeding of propionate and butyrate producers. Despite a lack of competitiveness against *Bifidobacterium* spp. such an early colonization might prime the intestinal microbial community for the development of degradation and fermentation capacity as soon as breastfeeding has been terminated. This is supported by the observation that fecal propionate and butyrate levels increased once breastfeeding was terminated, or even before weaning in the non-BF group.

### *Clostridiaceae* and *Peptostreptococcaceae* can selectively use HMO derived monomers or metabolites

Appert *et al*.^12^ identified members of the families *Clostridiaceae* and *Peptostreptococcaceae* as first butyrate producers in samples of 3-month-old infants. These families include taxa that are characterized by their ability of producing butyrate from the catabolism of other compounds such glucose or acetate, but also through peptidoolytic activity.^48^ Here, we show that strains of *C. perfringens, C. neonatale* and *C. difficile* utilized HMO derived fucose and 1,2-PD. A study performed by Salli *et al*. ^49^ observed a small increase in turbidity of *C. perfringens* E-98861 grown in the presence of 3-FL and fucose but no growth of different strains of *C. difficile. C. perfringens* ATCC 13124 did not utilize fucosyl- or sialyllactose.^39^ These results indicate that *Clostridiaceae* and *Peptostreptococcaceae* might profit from HMO derived cross-feeding in a species- and/or strain-dependent manner, which seems, however, not to contribute to the overall competitiveness especially of the *Peptostreptococcaceae* in breastfed infants during the first months of life.

### The fermentation metabolite lactate impacts *C. difficile* and *I. bartlettii* abundance and growth

At 3 months, we observed significantly higher levels of *Peptostreptococcaceae* in non-BF infants. Several publications agree on a lower occurrence of the family *Peptostreptococcaceae* when comparing breastfed and non-breastfed infants.^13,15,50,51^ Among the *Peptostreptococcaceae, C. difficile* abundance has been repeatedly related to a non-breastfeeding status pointing at a breastmilk component or fermentation metabolite with inhibitory activity toward *C. difficile*.^52^ Here, the family *Peptostreptococcaceae* was less abundant in lactate-rich feces of breastfed infants, and strains of the species *C. difficile* and *I. bartlettii* were inhibited by lactic acid *in vitro*. Together, these results suggest that fermentation derived lactate might play an inhibitory role toward members of the *Peptostreptococcaceae* through direct interaction as an antimicrobial in breastfed infants; such an antimicrobial effect was not evident in non-breastfed infants. *C. difficile* is considered a commensal in infants with a toxin burden of about 20%, and pediatric *C. difficile* infections have been reported.^53,54^ Intestinal HMO fermentation might be a contributing a defense mechanism to reduce the risk of such an infection.

Even in exclusively-BF infants that were colonized with *C. difficile*, fecal microbial or metabolic indicators more closely resembled non-breastfed infants including a higher α-diversity, a lower relative abundance of *Bifidobacteriaceae*, higher relative abundance of *Bacillota* and *Pseudomonata*, and higher fecal propionate and butyrate levels compared to non-colonized infants^55^ supporting the observation that a breastfeeding-related fermentation profile impacts *C. difficile* colonization. A recent study reported that the inclusion of 2-FL to infant formula in combination with the probiotic lactate producer *Limosilactobacillus reuteri* led to significantly lower abundance of *C. difficile in vivo*, which could be partly attributed to the potential of *L. reuteri* to produce lactate.^56^

### Lactic acid had a stronger inhibitory effect than acetic acid

Based on the ‘weak acid theory’, the antimicrobial activity of SCFAs and lactate relies on the degree of dissociation as mainly the undissociated, more hydrophobic molecule passes through the cell membrane to dissociate intracellularly.^23^ Antimicrobial activity of SCFAs and lactate is therefore linked to environmental pH and the dissociation constant (pKa) of the weak acid; a higher pKa relates to higher antimicrobial activity compared weak acids with a low pKa. With a pKa of 3.86, lactic acid is a stronger acid but a weaker antimicrobial than acetic acid (pKa 4.76). In agreement, previous studies targeting species such as *Penicillium* sp, *Fusarium graminearum, Aspergillus niger* and *Escherichia coli*. ^57,58^ showed higher antimicrobial activity of acetic acid than lactic acid. In this study conducted at controlled pH, lactic acid inhibited *C. difficile* and *I. bartlettii* more effectively than acetic acid indicating a compound specific mode of action and/or species dependent susceptibility beyond the weak acid theory.

Lactic acid levels could also be connected to fecal pH. A review including studies from 1926 to 2017, reported a trend for an increase in fecal pH during the last century, which concurrently related to higher presence of bacterial families including *Clostridiaceae* and *Peptostreptococcaceae*.^59^ *In vitro*, we observed lower turbidity of strains of *Peptostreptococcaceae* and *Clostridiaceae* after growth in medium with pH 5.5 compared to 6.2.

### Breast milk is a source of SCFA and lactate

In addition to being exposed to SCFA and lactate produced by intestinal microbial HMO fermentation, breast milk contained acetate, formate, butyrate, and lactate. There have been only a few studies that investigated SCFA and lactate content in breast milk. Stinson *et al*. ^4^ reported a median content of 47 μM acetate, 96 μM butyrate, 44 μM formate and 171 μM lactate in breast milk from 109 mothers collected at 1 months.^4,60^ While we also consistently recovered SCFA and lactate, levels were up to three-fold lower. Nevertheless, these results show that breast milk should be considered a source of fermentation metabolites for potential cross-feeding or with antimicrobial activity. With an estimated daily consumption of 700 mL at 4 months, there would be a daily uptake of 30 µmol acetate, 159.9 µmol butyrate and 10.2 µmol formate, and 108.4 µmol lactate, which could contribute to antimicrobial activity if not absorbed in the upper gastrointestinal tract.^61^ Limited data is available on human lactate absorption. In sheep, absorption of lactate depended on concentration and pH while in pigs, absorption behavior was more similar to glucose than to other SCFA.^62,63^ Butyrate absorption occurs mainly in the colonocytes in the proximal colon either through passive diffusion or by active transport mechanisms linked to ion exchange.^64^

### Study limitations

Despite of all the findings presented above, this study presents some limitations. Collection of milk was performed at 1 and 3 months to cover two stages of lactation, while fecal samples were collected at 3, 6 and 12 months. The non-BF and partially-BF groups included a lower number of children compared to the exclusively-BF group, and we may not have had enough statistical power to detect differences between the three groups. Furthermore, most children of the non-BF group were also exposed to some breastmilk before 3 months, which may have caused underestimation of the effect of breastfeeding on the fecal microbiota or metabolite profile by making the groups similar. However, at 3 months, when the fecal samples were analyzed, the proportion of breastfeeding among the children in the non-BF group was 11/14 (75.8%) and children in the non-BF group were mixed-breastfed for a median of 7.5 weeks. Additionally, information on the dose or frequency of formula milk consumption was not available for the children in the partially breastfed group. Thus, it was not possible to assess whether there is a dose–response relationship between formula or breastmilk exposure and fecal microbiota or metabolites.

## Conclusion

This study shows a major impact of breastfeeding on microbial community composition and fermentation activity before weaning. Exclusive breastfeeding promoted the accumulation of fecal metabolites from *Bifidobacterium-*HMO utilization such as fucose, 1,2-PD and lactate with the potential involvement of other bacterial families. While breastfeeding likely suppressed additional fermentation activity, butyrate and propionate producers were already present at 3 months of age setting stage for an increase in fermentation capacity when breast milk is replaced with infant formula, or for the onset of weaning.

Members of the *Clostridiaceae* and *Peptostreptococcaceae* utilized HMO derived fucose and 1,2-PD, nevertheless, there was higher abundance in *Peptostreptococcaceae* family in the non-breastfed infants compared to the exclusively-BF which might directly relate to the antimicrobial activity of the fermentation metabolite lactate. These observations suggest that breastfeeding not only directs fermentation activity but that the fermentation metabolites themselves impact the composition of the intestinal microbiota. The results obtained here might suggest targeted interventions to direct colonization of selective microbes during the first months of life.

## Materials and methods

### Materials and chemicals

All chemicals were purchased from Merck (Denmark) unless specified otherwise.

### Cohort and sample collection

Childhood AlleRgy, nutrition and Environment (CARE) is a birth cohort in which 69 infants and mothers from Switzerland were chosen for the following project between 2016 and 2019. Mothers were recruited at the cantonal and children’s hospitals in St. Gallen. Ethical approval was obtained from the Ethics Commission from Ostschweiz (EKSG 15/005), the Swiss ethic ID is: PB_2017–00243. All healthy babies born in the Cantonal Hospital St. Gallen with at least one parent who speaks German were considered eligible for the study and invited to participate after birth. Exclusion criteria were congenital abnormality, chronic lung disease, immune deficiency of the child, or autoimmune disease of the mother.

The CARE cohort monitored the breastfeeding of the participants over time and collected milk samples at the age of 1 month (±1 week, 22–37 days) and 3 months (3–4 months, 80–177 days). Data presented in the manuscript on breastfeeding status was based on weekly food diaries until the end of year 1. Milk samples were collected by the participants. Mothers were advised to collect milk between 7 am and 1 pm in the course of breastfeeding. Afterwards, 5 mL of milk sample was collected in a tube. Samples were sent overnight with a cooling pad to the laboratory, were aliquoted within the period of 24–48 hours and kept at −80°C until analysis. Infants were divided into three groups based on their breastfeeding status at exactly 4 months as exclusively breastfed (Exclusively-BF) (*n = 38*), partially breastfed (Partially-BF) (*n = 17*) or not breastfed (Non-BF) (*n = 14*). Fecal samples were collected at 3 (90–120 days), 6 (180–210 days) and 12 months (330–390 days) were transferred to the laboratory as described by Appert et *al*. ^12^

### Analysis of microbial composition and fecal fermentation metabolites based on breastfeeding status

To determine the impact of breast milk on fecal microbiota composition and fermentation metabolites, the 16S rRNA gene libraries, qPCR-based abundance data and the fecal fermentation metabolites collected by Appert *et al*.^12^ were re-analyzed depending on time and breastfeeding status of the infants. Methods are described in detail in Appert *et al*. ^12^ Briefly, 16S rRNA gene sequencing was performed by targeting the V4 hypervariable region using the primers 515F (5’-GTGCCAGCMGCCGCGG TAA-3’) and 806 R (5’-GGACTACHVGGGTWTCTAAT-3’) using an Illumina MiSeq platform with 250 × 2 read length by the usage of v2 chemistry followed by bioinformatic analysis as described.^12^ 16S rRNA gene sequences are available at NCBI’s SRA archive under BioProject with accession number PRJNA616703. qPCR assays were performed to determine total abundance of bacteria using a Roche LightCycle 480 System. Total and relative bacteria abundance data was combined for quantitative microbiota profiling.^12^ Fecal fermentation metabolites and fucose obtained with a water-based extraction method were analyzed using HPLC-RI.^12^

The number of fecal samples collected at the different time points (3, 6 and 12 months) depending on the breastfeeding status of the infants were at 3 months: Exclusively-BF *n* = 34, Partially-BF *n* = 16, Non-BF *n* = 13; at 6 months: Exclusively-BF *n* = 38, Partially-BF *n* = 17, Non-BF *n* = 14; at 12 months: Exclusively-BF *n* = 38, Partially-BF *n* = 17, Non-BF *n* = 14.

In this study, we calculated alpha-diversity at an initial subsampling of ASVs higher than 15.000 reads or more, and further used to calculate the richness and diversity indices (Chao1, Shannon). Beta-diversity was calculated with all the entire gene catalog using Bray-Curtis dissimilarity matrix using the R package ‘vegan 2.6–2’. Principal Coordinate Analysis (PCoA) ordination was used for visualization, components that plot close present lower dissimilarity. Here, we combined the SILVA (v. 132) based assignment with additional BLASTn analysis (refseq_rna) for taxonomic identification to determine the species distribution within the ASV assigned to *Clostridiaceae* and *Peptostreptococcaceae* at 3 months.

### Strains and growth conditions

Strains (Suppl. Table S4) were obtained from the German Collection of Microorganisms and Cell Cultures (DSMZ, Germany), from the strain collection of the Department of Functional Microbe Technology (FMT) (Aarhus University, Denmark) or from the Department of Microbiology, Dietetics and Nutrition of Czech University of Life Sciences Prague (CZU, Czechia). Bacteria were routinely cultivated in Wilkins-Chalgren supplemented with 5 g/L soya peptone (WCSP, Biolife), 1 g/L Tween 80, and 0.5 g/L L-cysteine-HCl. All components were solubilized in milliQ water. The pH was adjusted to 7.1 before boiling, and L-cysteine-HCl was added before degassing for a final pH of 6.5 after autoclaving. Samples were dispersed in Hungate tubes under constant CO_2_ flow and were autoclaved at 120°C for 20 min. To reactive strains from −80°C glycerol stocks and to generate working cultures, strains were streaked on anaerobically prepared WCSP agar plates (WSCP with the addition of 15 g/L agar (VWR)) in an anaerobic chamber (10% CO_2_, 5% H_2_, 85% N_2_, Coy Laboratories, USA) and were incubated for 3 days. Single colonies were picked and inoculated in anaerobic WCSP broth in Hungate tubes at 37°C for 24 h unless indicated otherwise. Strain identification was confirmed by Sanger sequencing of the 16S rRNA gene as indicated in Suppl. Table S4. Strain purity was routinely checked using microscopy.

### Utilization of fucose, HMO and selected fermentation metabolites by strains of *Clostridiaceae* and *Peptostreptococcaceae*

To test the utilization of fucose, HMO and fermentation metabolites, single colonies from WCSP agar were transferred to a Hungate tube containing anaerobically prepared, modified yeast extract-casitone (YC) medium that was prepared as described by Duncan *et al*. ^27^ without volatile fatty acids but with 51 mM glucose (YC-glucose). After 24 h incubation at 37°C, 2% was transferred to new tubes with YC-glucose for incubation at 37°C for 24 h. Consequently, each strain was transferred (2%) to YC containing glucose (YC-glucose, positive control), YC with lactic acid (55 mM, YC-lactate), YC with 1,2-PD (43 mM, YC-1,2-PD); YC with fucose (45 mM) (YC-fuc). YC without additional carbon source as background control (YC). Turbidity was measured with the McFarland Densitometer (Grant-Bio) at 0 and 24 h of incubation. At 0 and 24 h, 1 mL culture was centrifuged at 10 000 g for 4 min for fermentation metabolite analysis. All experimental work was performed in at least independent triplicates.

### Antimicrobial activity of lactic and acetic acid

The antibacterial activity of lactic and acetic acid was tested using two-fold broth dilution assay in 96-well sterile microtiter plates essentially as described.^65^ Briefly, a two-fold dilution series of the organic acids was prepared in an anaerobic chamber in microtiter plates filled with 100 μL anaerobically prepared WCSP adjusted to pH 5.5 or 6.2. The maximum concentration of lactic and acetic acid was 100 mM. The cooperative antibacterial activity of lactic and acetic acid against *C. perfringens* FMT 568 and *C. difficile* DSM 12056 was tested using a modified broth dilution assay in 96-well sterile microtiter plates (VWR). Two-fold broth dilution assays were prepared horizontally (acetic acid) and vertically (lactic acid) with highest concentrations of 50 mM. Cells from overnight cultures of selected *Clostridiaceae* and *Peptostreptococcaceae* (Suppl. Table S4) grown in WSCP broth were diluted 100-fold in fresh WCSP at the respective pH and were added at 10%. Plates were incubated in an anaerobic bench at 37°C for 24 h. Positive controls (cultivation medium without organic acid), as well as blanks (sterile media instead of cell suspension), were included in each assay. Bacterial growth was detected by measuring the optical density at 590 nm after 24 h of incubation using the Infinite M200 Pro Plate Reader from TECAN. A sigmoidal (four parameter logistic) equation was used to fit the data (Sigma Plot version 15, Alfasoft); the inflection point of the resulting curve represents the MIC_50_ value, which was defined as the concentration that reduced final optical density of the test strains to 50% compared to optical density without inhibitors. Every strain was analyzed at least 3 times unless otherwise indicated.

### Carbohydrate and metabolite analysis by HPLC-RI

Fermentation metabolites were measured from the supernatants obtained from the different YC-media at 24 h using the 1260 Infinity II LC System equipped with a Hi-Plex H guard (7.7 × 50 mm, 8 µm) and separation (300 × 7.7 mm) columns and a refractive index detector (all Agilent). The samples (10 μL injection volume) were eluted 5 mM H_2_SO_4_ at a flow rate of 0.6 mL min^−1^ at 40°C. Fucose and fermentation metabolites were quantified using external standards and Chromeleon Console (version 7.7.2.10) for analysis.

### Analysis of the milk samples by ^1^H-NMR spectroscopy

In total 84 milk samples (At 1 month: Exclusively-BF *n* = 16, Partially-BF *n* = 7, Non-BF *n* = 7; at 3 months: Exclusively-BF *n* = 31, Partially-BF *n* = 12) were analyzed using ^1^H-NMR was performed as described.^66^ Briefly, samples were removed from the −80°C, were thawed at room temperature for 30 min and were transferred to ice. Milk samples (300 μL) were transferred to a microcentrifuge tube, 300 μL milliQ water was added, vortexed and centrifuged at 4 000 × g for 10 min at 4°C, in order to skim the milk. Filters (Amicon Ultra 0.5 centrifugal filter 10K) were washed with 500 µL milliQ water three times by centrifugation at 10 000 × g for 10 min at 4°C followed by drying (filters inverted) by centrifugation at 800 × g for 10 sec at 4°C. After scooping the fat layer off the milk samples, 500 μL milk were added to the dry filters and centrifuged at 10 000 × g for 60 min at 4°C. The filtration product (400 μL) was transferred to a new microcentrifuge followed by the addition of 200 μL of a mixture of 60 µL deuterium oxide (D_2_O) containing 0.05% trimethylsilylpropanoic acid as internal chemical shift reference, and 140 µL milliQ water. The mixture (600 μL) was transferred then to an NMR tube (NMR 4’’ Tubes 5 mm, Bruker). NMR spectroscopy was performed on a 600 Bruker Neo-IVDR NMR spectrometer, operating at a ^1^H frequency of 600.03 MHz, and equipped with a 5-mm ^1^H BBI probe (Bruker). All ^1^H spectra were referenced to the TSP signal at 0 ppm. ^1^H-NMR spectra were phase and baseline corrected manually using Topspin 4.09 (Bruker Biospin). NMR signals were assigned in accordance with previous literature, 2D NMR spectroscopy, Chenomx NMR Suite 8.6 (Chenomx Inc) and the Human Metabolome Database.

### Statistical analysis

Data analysis and statistics were performed by R Studio with the packages FSA, ggplot2, RcolorBrewer, ggpubr (Version 1.3.1093). Normality was tested for the metabolites determined by ^1^H-NMR by Shapiro-Wilk’s method and was identified as not normally distributed (*p < 0.05*) similar to the data provided by Appert *et al*.^12^ Kruskal–Wallis test was used for the univariate comparison and Dunn test for the multiple posthoc comparisons with p-values adjusted with the Holm method using the package ‘rstatix’ version 0.7.0. Pairwise comparisons using Wilcoxon rank sum exact test and adjustment by Holm method was used for adjustment of p-values after Kruskal–Wallis test for pair-wise comparisons using the package ‘rstatix’ version 0.7.0. Chi square test was used for the categorical comparison using the package ‘rstatix’ version 0.7.0. Vegan package was used for calculations of beta diversity and Permutational multivariate analysis of variance (PERMANOVA).

Factor analysis for mixed data (FAMD) included categorical variables such as delivery mode, secretor phenotype, breastfeeding status and quantitative data such as absolute abundance of the families derived from quantitative microbiota profiling, fecal fermented (f_) metabolites and breastmilk (bm_) metabolites at 3 months. The values around the coordinate 0 reflect variables with low power of discrimination, therefore they do not influence in the distinction of the groups. Variables closer to the −1 or 1 coordinates have high power of discrimination. In this study, values with a contribution equal or below 1 in both dimensions 1 and 2 were discarded from the plotting.

Figures were made with RStudio (Version 1.3.1093), BioRender, Canva or with Sigma Plot.

## Supplementary Material

Supplemental Material

## Data Availability

The authors confirm that the data supporting the findings of this study are either submitted to public repository (PRJNA616703) or available within the article and its supplementary materials.

## Appendix

Thomas Bieber^1,2^; Peter Schmid-Grendelmeier^1,3^; Claudia Traidl-Hoffmann^1,4,5^; Marie-Charlotte Brüggen^1,3,6,7^; Claudio Rhyner^1,2,8^

^1^Christine Kühne-Center for Allergy Research and Education Davos (CK-CARE), Davos, Switzerland

^2^Davos Biosciences, Davos, Switzerland

^3^Allergy Unit, Department of Dermatology, University Hospital of Zürich, Zürich, Switzerland

^4^Environmental Medicine, Faculty of Medicine, University of Augsburg, Augsburg, Germany

^5^Institute of Environmental Medicine, Helmholtz Zentrum Muenchen, German Research Center for Environmental Health, Augsburg, Germany

^6^Hochgebirgsklinik Davos, Davos, Switzerland

^7^Faculty of Medicine, University of Zürich, Zürich, Switzerland

^8^Swiss Institute of Allergy and Asthma Research (SIAF), Davos, Switzerland
