# Supplementary material for: Breastfeeding and the major fermentation metabolite lactate determine occurrence of Peptostreptococcaceae in infant feces
Source: Gut Microbes. 2023 Aug 18;15(1):2241209. doi: 10.1080/19490976.2023.2241209 (PMC10449005; doi:10.1080/19490976.2023.2241209)
Supplement: Supplemental Material [file KGMI_A_2241209_SM2142.docx]

# SUPPLEMENTARY FILE FOR

**Breastfeeding and the major fermentation metabolite lactate determine occurrence of *Peptostreptococcaceae* in infant feces**

Lucía Huertas-Díaz^a^, Rikke Kyhnau^b^, Eugenio Ingribelli^c^, Vera Neuzil-Bunesova^c^, Qing Li^a^, Mari Sasaki^d^, Roger P. Lauener^e,f^, Caroline Roduit^d,e,f^, Remo Frei^e,g^, CK-CARE study group^#^, Ulrik Sundekilde^b^, Clarissa Schwab^a*^

^a^Department of Biological and Chemical Engineering, Aarhus University, Aarhus, Denmark, ^b^Department of Food Science, Agro Food Park 48, Aarhus University, Aarhus, Denmark, ^c^Department of Microbiology, Nutrition and Dietetics, Czech University of Life Sciences Prague, Prague, Czech Republic, ^d^University Children’s Hospital Zürich, Zürich, Switzerland, ^e^Christine Kühne-Center for Allergy Research and Education (CK-CARE), Davos, Switzerland, ^f^Children’s Hospital St. Gallen, St. Gallen, Switzerland, ^g^Division of Respiratory Medicine, Department of Paediatrics, Inselspital, University of Bern, Bern, Switzerland

^#^Appendix A

*corresponding author. Email: schwab@bce.au.dk

**TABLE S1.** **Median of the Human Milk Oligosaccharides (HMOs) concentration from breastmilk.** Infants were group based on in exclusively-, partially- or non-breastfed (BF) infants depending on the breastfeeding status at 4 months, which is the minimum length of exclusive breastfeeding recommended in Switzerland. Samples were collected at two time points (1 and 3 months), and infants were classified based on breastfeeding status as exclusively breastfed. partially or non-breastfed**.** Major HMOs were analysed using NMR. 2-FL: 2-Fucosyllactose; 3-FL: 3-Fucosyllactose; LDFT: lactodifucotetraose; LDNFH I: Lacto-N-difucohexaose I; LDNFH II: Lacto-N-difucohexaose II; LNFP I: Lacto-N-fucopentaose I; LNFP V: Lacto-N-fucopentaose I; DSLNT: disialyllacto-N-tetraose; 3-SL: 3-Sialyllactose; 6-SL: 6-Sialyllactose. BF: breasted; n: number of samples in each group; IQR: interquartile range (Q1-Q3); n.s: no significance; no significance between occurrence were found. f: frequency of milk samples presenting the oligosaccharide. NA: non-available data.

| Concentration (median, interquartile range (Q1-Q3), μM) | | | | | | | | | | | | | |
| --- | --- | --- | --- | --- | --- | --- | --- | --- | --- | --- | --- | --- | --- |
| 1 Month | | | | | | | **p-value** | **3 Months** | | | | | **p-value** |
| HMO | **Exclusively-BF** | **f** | **Non-BF** | **f** | **Partially-BF** | **f** |  | **Exclusively-BF** | **f** | **Non-BF** | **Partially-BF** | **f** |  |
| 2-FL | 2771.2 (2438.4-2936.8) | 12/16 | 2554.9 (1720.6-3746.8) | 5/7 | 3210.1 (2272.8-3743.4) | 6/7 | n.s | 2071.7 (1874.1-2979.6) | 25/31 | NA | 1579.1 (1245.3-2375) | 11/12 | n.s |
| 3-FL | 1156.8 (806.8-2140.9) | 16/16 | 1706.8 (1543.7-1974.9) | 7/7 | 1168.9 (867.7-1630) | 7/7 | n.s | 1856.9 (957.3-2694) | 31/31 | NA | 2023.4 (1201.4-3165) | 12/12 | n.s |
| LDFT | 455.1 (335.5-566.9) | 12/16 | 2349.1 (994.1-3725.5) | 4/7 | 430 (355.6-951.9) | 7/7 | n.s | 503.6 (360.7-638.9) | 25/31 | NA | 429.3 (358.9-530.1) | 11/12 | n.s |
| LNDFH I | 1097.7 (816.1-1341.3) | 10/16 | 1375.3 (663.5-1760.1) | 6/7 | 1209 (965-1342.8) | 6/7 | n.s | 566.3 (476.2-646.5) | 21/31 | NA | 687.9 (506.6-824.1) | 10/12 | n.s |
| LNDFH II | 242.9 (183.9-949.3) | 14/16 | 481.3 (390.6-551.4) | 6/7 | 266.3 (159.6-303.8) | 7/7 | n.s | 212.7 (175-284.5) | 24/31 | NA | 293.7 (143.4-336.4) | 11/12 | n.s |
| LNFPI | 449.8 (346.7-501.9) | 16/16 | 446.8 (286.6-473.7) | 7/7 | 457.1 (349.3-577.2) | 7/7 | n.s | 143.6 (106.3-194.2) | 31/31 | NA | 169.1 (119.5-205.5) | 12/12 | n.s |
| LNFPV | 257.1 (177.9-350.3) | 15/16 | 359.9 (319.8-530.6) | 6/7 | 327.7 (206.1-410.9) | 7/7 | n.s | 609.7 (538-737.9) | 30/31 | NA | 626.8 (545.2-789.5) | 12/12 | n.s |
| DSLNT | 202.6 (164.1-249.5) | 16/16 | 303.9 (195-366) | 7/7 | 208.9 (147.2-247.3) | 7/7 | n.s | 82.2 (67.8-94.4) | 31/31 | NA | 74.6 (65.5-108.6) | 12/12 | n.s |
| 3-SL | 193.1 (167.8-210.5) | 16/16 | 208.6 (181-229.4) | 7/7 | 155.1 (135-160.7) | 7/7 | * | 142.2 (123.1-171.1) | 31/31 | NA | 128.7 (117.2-138.4) | 12/12 | n.s |
| 6-SL | 83.3 (71.3-122.8) | 16/16 | 93.4 (74.6-101.8) | 7/7 | 75.2 (65.8-91.9) | 7/7 | n.s | 26.4 (16.8-37.5) | 31/31 | NA | 24.4 (15.4-32.9) | 12/12 | n.s |
| Lactose | 125214.9 (117684.4-132616.7) | 16/16 | 122537.6 (110212.1-129790.7) | 7/7 | 136027 (121519.5-139662.1) | 7/7 | n.s | 135729.3 (127731.6-142529.3) | 31/31 | NA | 135473.7 (129578.5-138328.3) | 12/12 | n.s |
| *n* |  | *16* |  | *7* |  | *7* |  |  | *31* |  |  | *12* |  |

**TABLE S2.** **Median of the Short Chain Fatty Acids (SCFA), lactate and lactose concentration from mother’s breastmilk samples**. Infants were group based on in exclusively-, partially- or non-breastfed (BF) infants depending on the breastfeeding status at 4 months, which is the minimum length of exclusive breastfeeding recommended in Switzerland. SCFA (μM) were analysed at two different time points (1 and 3 months) using NMR, and samples were classified by their infant’s breastfeeding status (exclusively breastfed. partially or non-breastfed). BF: breasted; n: number of samples in each group; IQR: interquartile range (Q1-Q3); n.s: no significance; no significance between occurrence were found. f: frequency of milk samples presenting the metabolite. NA: non-available data.

| Concentration (median, interquartile range (Q1-Q3), μM) | | | | | | | | | | | | | | |
| --- | --- | --- | --- | --- | --- | --- | --- | --- | --- | --- | --- | --- | --- | --- |
| 1 Month | | | | | | | **p-value** | **3 Months** | | | | | **p-value** | |
| Breast milk component | **Exclusively-BF** | **f** | **Non-BF** | **f** | **Partially-BF** | **f** |  | **Exclusively-BF** | **f** | **Non-BF** | **Partially-BF** | **f** |  |  |
| Acetate | 18.8 (11.9-45.3) | 16/16 | 90.4 (13-205.8) | 7/7 | 30.7 (19.8-41.3) | 7/7 | n.s | 43.2 (23.1-124.2) | 31/31 | NA | 42.1 (23.9-83.3) | 12/12 | n.s |  |
| Butyrate | 67.7 (48.3-132.6) | 16/16 | 36.3 (34.3-70.3) | 7/7 | 68.3 (36.3-101.1) | 7/7 | n.s | 159.4 (83-297) | 31/31 | NA | 308.6 (190.3-463) | 12/12 | n.s |  |
| Formate | 15.2 (14.1-18.2) | 12/16 | 19.5 (16-41.5) | 6/7 | 13.2 (12.9-20.5) | 5/7 | n.s | 14.5 (12.9-20.2) | 24/31 | NA | 13.2 (11.8-23.3) | 8/12 | n.s |  |
| Lactate | 104.8 (81-244.8) | 16/16 | 113.5 (107.1-160.1) | 7/7 | 207.9 (186.6-635.7) | 7/7 | n.s | 168.3 (104.9-276) | 31/31 | NA | 104.4 (50-246.8) | 12/12 | n.s |  |
| *n* | *16* |  |  | *7* |  | *7* |  |  | *31* | *-* |  | *12* |  |  |

**TABLE S3. Occurrence in percentage of different metabolites in feces at different time points and depending on breastfeeding status**. Significant differences with Dunn test for the multivariate comparisons with p-values adjusted with the Holm method. Different letters within each breastfeeding group at a specific time point denote significant differences (p<0.05). BF: Breastfed. n = number of infants.

|  | Occurrence (%) | | | | | | | | |
| --- | --- | --- | --- | --- | --- | --- | --- | --- | --- |
|  | 3 Months | | | 6 Months | | | 12 Months | | |
|  | **Exclusively-BF** | **Partially-BF** | **Non-BF** | **Exclusively-BF** | **Partially-BF** | **Non-BF** | **Exclusively-BF** | **Partially-BF** | **Non-BF** |
| Acetate | 100 | 100 | 92.3 | 100 | 94.1 | 100 | 100 | 88.2 | 100 |
| Propionate | 41.2 | 62.5 | 92.3 | 84.2 | 82.4 | 100 | 100 | 88.2 | 92.9 |
| Butyrate | 20.6 | 43.8 | 61.5 | 57.9 | 71.4 | 57.9 | 94.7 | 82.4 | 85.7 |
| Lactate | 88.2 | 81.2 | 61.5 | 78.9 | 64.7 | 71.4 | 44.7 | 41.2 | 42.9 |
| Fucose | 100 | 100 | 92.3 | 97.4 | 82.4 | 92.9 | 86.8 | 82.4 | 64.3 |
| 1,2PD | 82.4 | 62.5 | 23.1 | 39.5 | 52.9 | 14.3 | 31.6 | 29.4 | 35.7 |
| n | 34 | 16 | 13 | 38 | 17 | 14 | 38 | 17 | 14 |

**TABLE S4. Most abundant *Peptostreptococcaceae and Clostridiaceae*.** ASVs from the 16S-rRNA in the feces at 3 months from the *Peptostreptococcaceae* family and from the *Clostridiaceae_1* family, from which representative strains were selected for the *in vitro* experiments, NA, not applicable

| Family | Genus | species | Representation of species within the family (%) | Strain ID | Origin | Identification method | Homology (%) |
| --- | --- | --- | --- | --- | --- | --- | --- |
| *Peptostreptococcaceae* | *Clostridioides* | *difficile* | 81.7% | FMT 1007  DSM 12056 | Infant feces  Rumen of new-born lamb | 16S rRNA  16S rRNA | 1076/1078 (99%)  974/980 (99%) |
| *Peptostreptococcaceae* | *Intestinibacter* | *bartlettii* | 13.9% | DSM 16795 | Human fecal material | NA |  |
| *Clostridiaceae_1* | *Clostridium_sensu_stricto_1* | *perfringens* | 61.9% | FMT 1006  FMT 568 | Infant feces  Fecal sample of Easten Bongo 23 G1 | 16S rRNA  16S rRNA | 915/923 (99%)  397/405 (98%) |
| *Clostridiaceae_1* | *Clostridium_sensu_stricto_1* | *butyricum* | 16.3% | CBL 4 | Infant feces | 16S rRNA | 747/758 (99%) |
| *Clostridiaceae_1* | *Clostridium_sensu_stricto_1* | *paraputrificum* | 10.5% | DSM 103826 | Human feces |  |  |
| *Clostridiaceae_1* | *Clostridium_sensu_stricto_1* | *baratii* | 1.3% | FMT 558 | Fecal sample of chilean flamingo 34 G1 & 34 G2 | 16S rRNA | 880/892 (99%) |
| *Clostridiaceae_1* | *Clostridium_sensu_stricto_1* | *neonatale* | 0.5% | LiF | Infant feces |  |  |

**
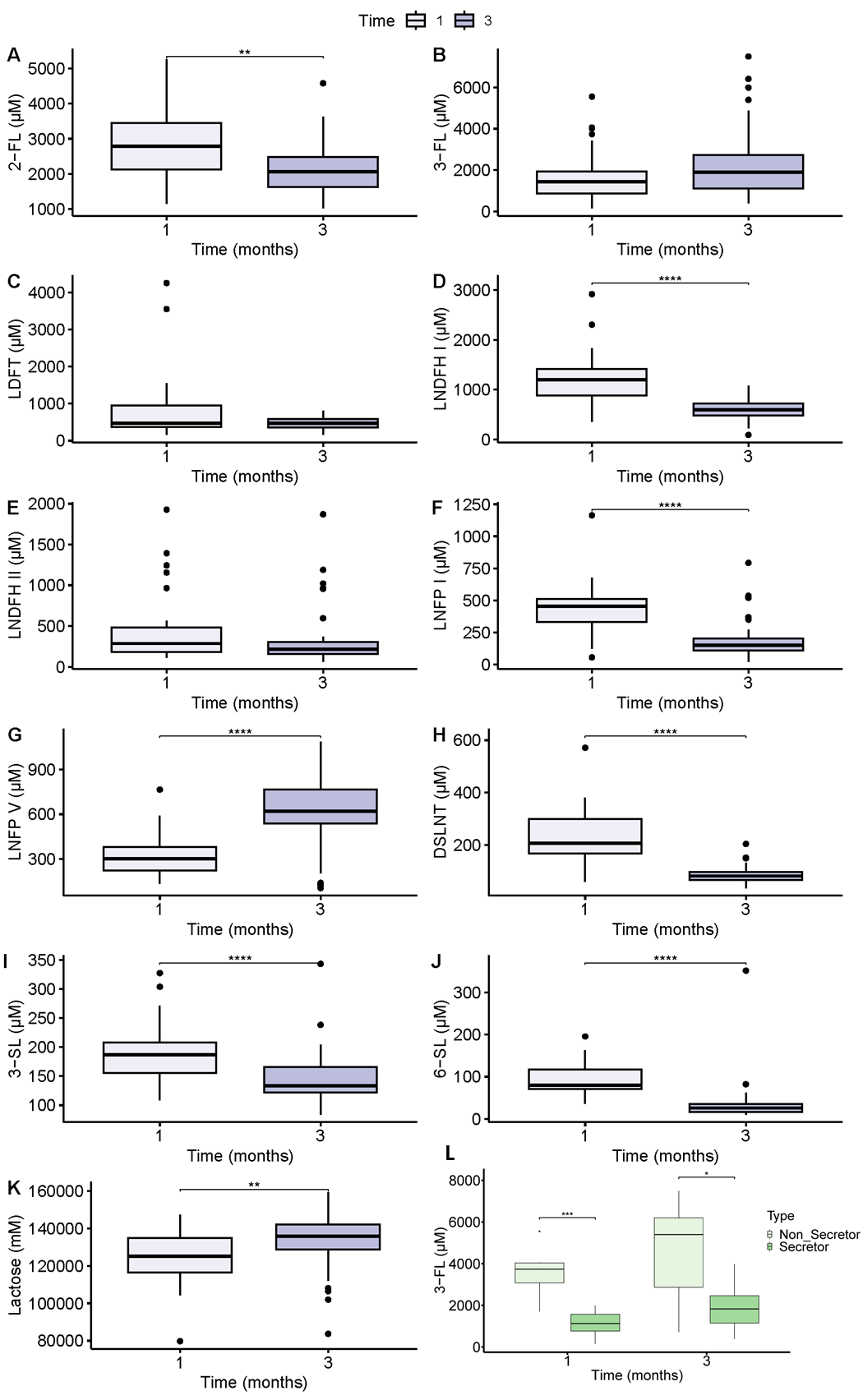
**

**FIGURE S1. Levels of major HMOs at 1 and 3 months and in different secretor status from breastmilk samples**. Metabolites shown in concentration (μM). 2-FL: 2-Fucosyllactose; 3-FL: 3-Fucosyllactose; LDFT: lactodifucotetraose; LDNFH I: Lacto-N-difucohexaose I; LDNFH II: Lacto-N-difucohexaose II; LNFP I: Lacto-N-fucopentaose I; LNFP V: Lacto-N-fucopentaose V; DSLNT: disialyllacto-N-tetraose; 3-SL: 3- Sialyllactose; 6-SL: 6-Sialyllactose. Significant differences shown: ‘****’ 0, ‘***’ 0.001 ‘**’ 0.01 ‘*’ 0.05. Pairwise comparisons using Dunn test and adjustment by Holm method.


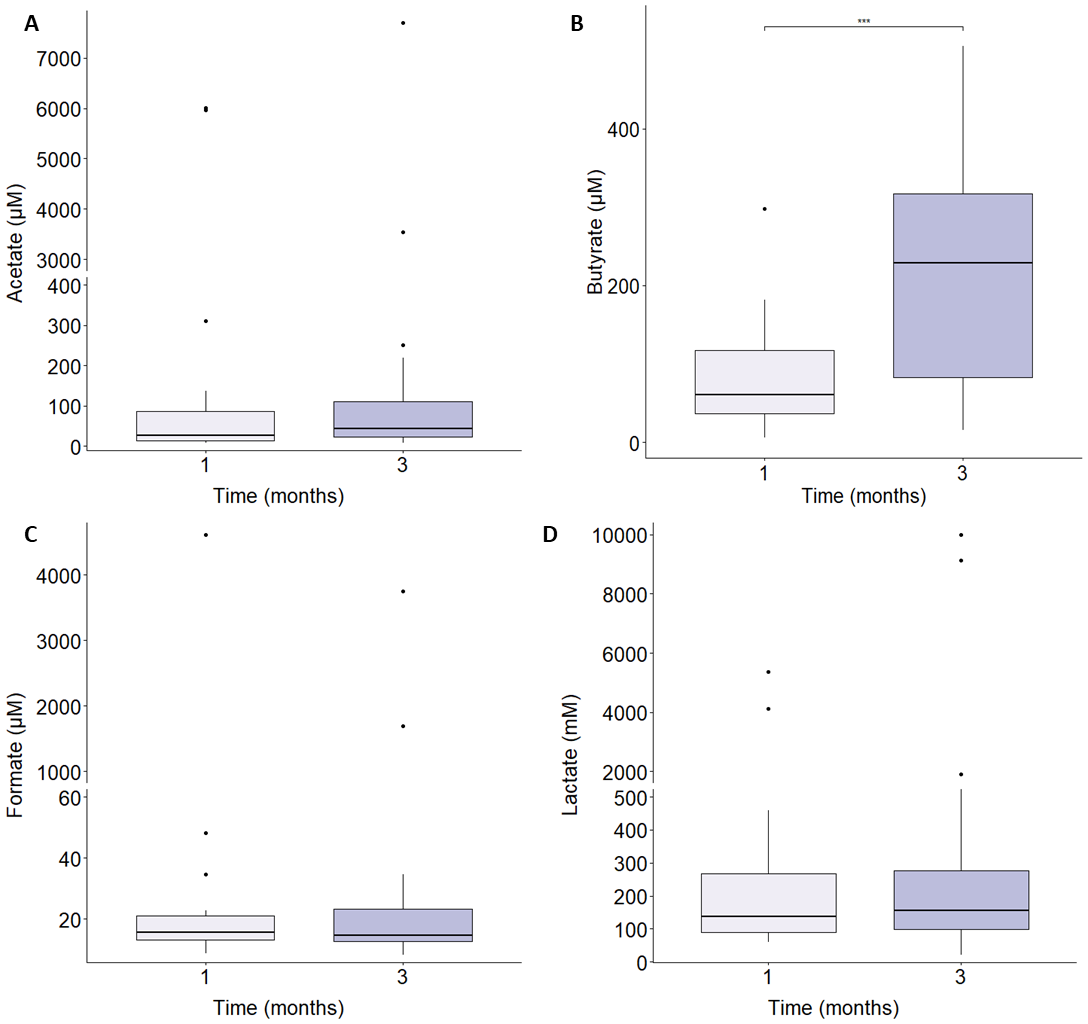


**FIGURE S2. Short Chain Fatty Acids (SCFA) and lactose concentrations (μM) from breastmilk samples at two different time points (1 and 3 months).** Significant differences shown: ‘****’ 0, ‘***’ 0.001 ‘**’ 0.01 ‘*’ 0.05. Pairwise comparisons using Dunn test and adjustment by Holm method.


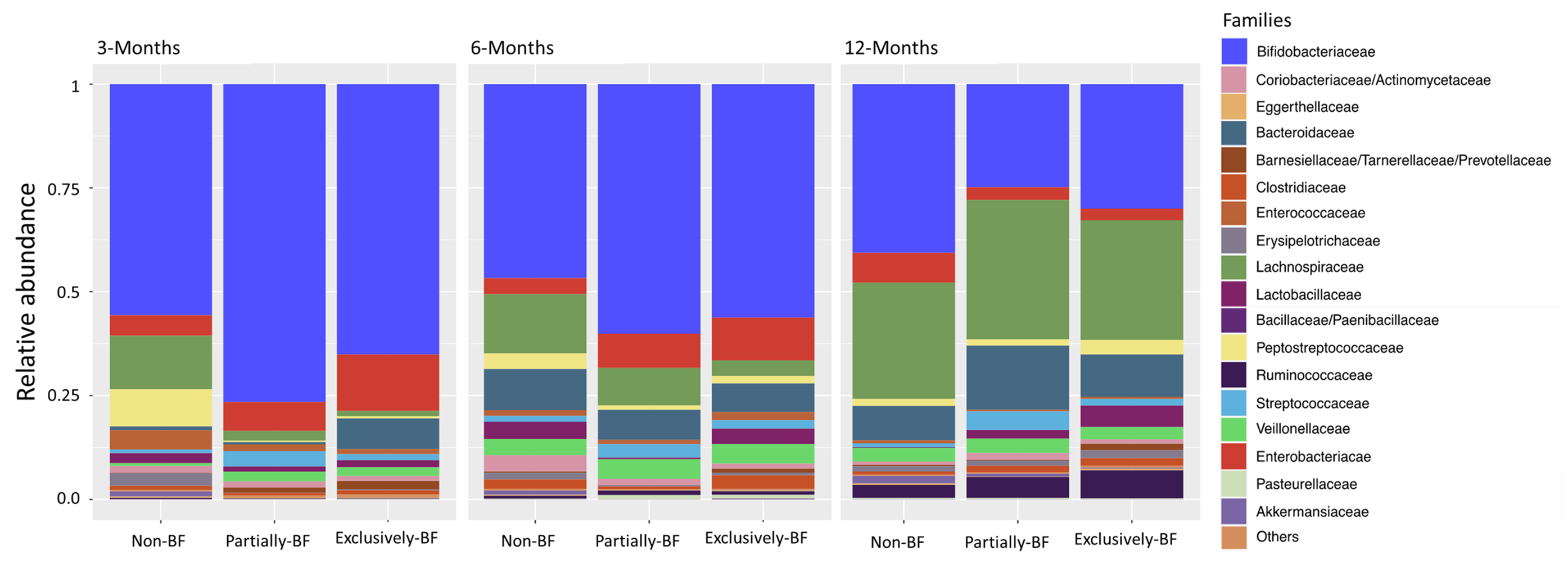


**FIGURE S3. Relative abundance of families through the different time points (3, 6 and 12 months) depending on different breastfeeding status**. Data was calculated from the 16S rRNA reads by the summation of reads within each family and further calculation of proportions. BF: breastfed.


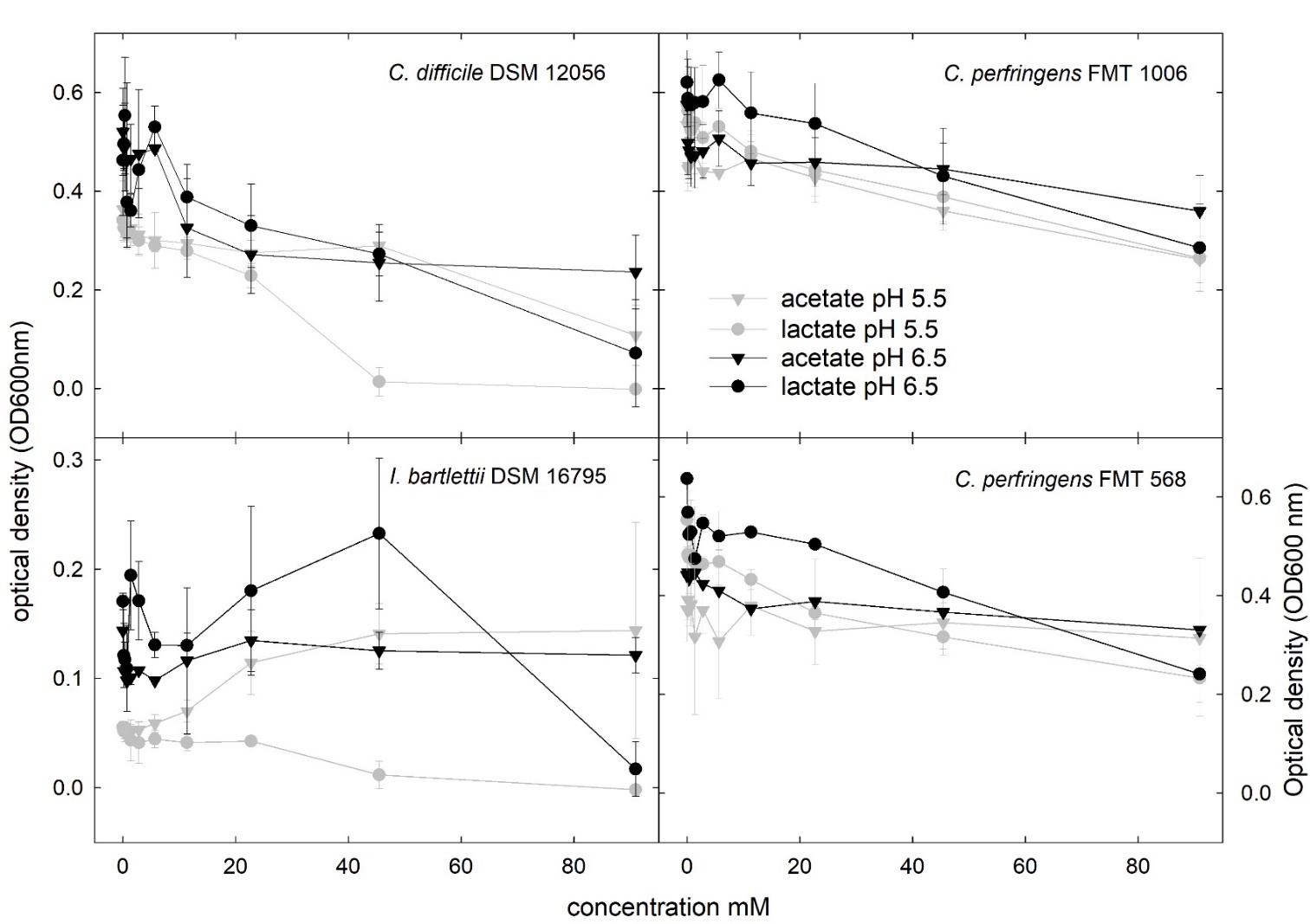


**FIGURE S4. Inhibition of selected Clostridiaceae and Peptostreptococcacaceae by lactic acid and acetic acid**. Two-fold dilution assay were performed in 96 well microtiter plates at pH 5.5 and 6.2 using C. difficile DSM 12056, I. bartlettii DSM 16795, C. perfringens FMT 568 and FMT 1006 as indicator strains. Optical density (OD600nm) was determined after 24 h incubation at 37°C at anaerobic conditions.
